# Biocompatible Multifunctional Polymeric Material for Mineralized Tissue Adhesion

**DOI:** 10.1002/adhm.202501993

**Published:** 2025-08-18

**Authors:** Yan Luo, Chenyang Zhang, Sage Fulco, Jingyi Liu, Keyu Chen, Yuntao Hu, Yuchen Jiang, Rui Xu, Leela Rakesh, Ozer Fusun, Ottman Tertuliano, Kevin Turner, Kyle H. Vining

**Affiliations:** ^1^ Mechanical Engineering and Applied Mechanics School of Engineering and Applied Science University of Pennsylvania Philadelphia PA 19104 USA; ^2^ Materials Science and Engineering School of Engineering and Applied Science University of Pennsylvania Philadelphia PA 19104 USA; ^3^ Bioengineering School of Engineering and Applied Science University of Pennsylvania Philadelphia PA 19104 USA; ^4^ Robotics School of Engineering and Applied Science University of Pennsylvania Philadelphia PA 19104 USA; ^5^ Computer and Information Science School of Engineering and Applied Science University of Pennsylvania Philadelphia PA 19104 USA; ^6^ Biomedical Engineering School of Engineering Vanderbilt University Nashville TN 37235‐1631 USA; ^7^ Mathematics Center for Applied Mathematics and Polymer Fluid Dynamics College of Science and Engineering Central Michigan University Mount Pleasant MI 48859 USA; ^8^ Preventive and Restorative Dentistry School of Dental Medicine University of Pennsylvania Philadelphia PA 19104 USA; ^9^ Center for Innovation and Precision Dentistry University of Pennsylvania Philadelphia PA 19104 USA

**Keywords:** dentin adhesion, molecular dynamics simulation, nanoindentation, thiol‐ene polymerization, triacrylate resin

## Abstract

This study develops a biocompatible multifunctional thiol‐ene resin system for adhesion to dentin mineralized tissue. Adhesive resins maintain the strength and longevity of dental composite restorations through chemophysical bonding to exposed dentin surfaces after cavity preparations. Monomers of conventional adhesive systems may result in inhomogeneous polymer networks and the release of residual monomers that cause cytotoxicity. In this study, a one‐step multifunctional polymeric resin system by incorporating trimethylolpropane triacrylate (TMPTA) and bis[2‐(methacryloyloxy)ethyl] phosphate (BMEP) is developed to enhance both mechanical properties and adhesion to dentin. Molecular dynamics simulations identify an optimal triacylate:trithiol ratio of 2.5:1, which is consistent with rheological and mechanical tests that yield a storage modulus of ≈30 MPa with or without BMEP. Shear bond tests demonstrate that the addition of BMEP significantly improves dentin adhesion, achieving a shear bond strength of 10.8 MPa, comparable to the commercial primer Clearfil SE Bond. Nanoindentation modulus mapping characterizes the hybrid layer and mechanical gradient of the adhesive resin system. Further, the triacrylate‐BMEP resin shows biocompatibility with dental pulp cells and fibroblasts in vitro. These findings suggest that the triacrylate‐trithiol crosslinking and chemophysical bonding of BMEP provide enhanced bond strength and biocompatibility for dental applications.

## Graphical abstract

A multifunctional thiol‐ene resin system is developed that provides a strong adhesive interface with dentin while maintaining biocompatibility with dental pulp cells. The addition of an acidic monomer to a triacrylate‐trithiol‐based resin achieves over 10 MPa shear bond strength by reinforcing the hybrid interface. The resin material shows promise for bioactive approaches, such as remineralization and localized drug delivery.

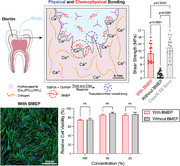

## 1 Introduction

Clinical applications involving dental tissues require polymeric adhesives for etching and bonding to mineralized tissues. In restorative dentistry, failed restorations and tooth fractures remain a significant problem despite advancements in dentin resin adhesive materials.^[^ ^1^, ^2^, ^3^, ^4^ ^]^ Further, residual monomers released from adhesive materials can negatively impact surrounding biological tissues.^[^ ^5^, ^6^ ^]^ There remains a need for biocompatible polymeric materials that can achieve strong adhesion to mineralized tissues.

Dental decay is increasing in prevalence, with over 3 billion untreated cases, despite increased access to prevention and dental treatments worldwide.^[^ ^7^ ^]^ Dental decay is treated by removing the decayed tooth material, preparing the tooth for direct access to dentin, and placing a direct resin restoration with a bonded adhesive resin interface.^[^ ^8^ ^]^ Adhesion to the dentin protects the restoration from microleakage, tooth fracture, infection, and ultimate failure of the treatment.^[^ ^9^ ^]^ The widely used commercial methacrylate‐based adhesive, Clearfil SE Bond, shows ≈12 MPa shear bond strengths.^[^ ^10^, ^11^ ^]^ While some studies report double bond conversion rates above 80%,^[^ ^12^ ^]^ others indicate only 56% double bond conversion for the methacrylate functional groups.^[^ ^13^ ^]^ Lack of conversion may lead to inhomogeneous polymer networks, residual monomers in the resin, and cytotoxicity and disruption of cellular signaling.^[^ ^5^, ^14^ ^]^ Hydrolytic degradation and water sorption compromise the long‐term durability of adhesive resins.^[^ ^14^, ^15^, ^16^ ^]^ Acrylamide‐based polymers and thiol‐ene systems have emerged as promising alternatives for dentin bonding, offering improved resistance to hydrolytic and enzymatic degradation,^[^ ^17^, ^18^ ^]^ rapid curing, and low cytotoxicity.^[^ ^19^ ^]^

Thiol‐ene‐based polymeric materials can also achieve mechanical strength comparable to methacrylate and polyurethane systems while minimizing cytotoxic effects.^[^ ^19^ ^]^ Trimethylolpropane triacrylate (TMPTA) and structurally related trithiol (TMPTMP) monomers are investigated as three‐armed thiol‐ene crosslinked polymers for a biocompatible resin.^[^ ^14^ ^]^ Three acrylate groups in TMPTA provide a high degree of crosslinking, which improves thermal stability, mechanical strength, and glass transition temperature in UV‐curable systems.^[^ ^20^, ^21^ ^]^ TMPTA also enhances rheological properties like shear viscosity and storage modulus in various polymer applications, offering fast curing times and improved material hardness, making it more efficient than other monomers.^[^ ^22^, ^23^ ^]^ Thiol‐ene materials crosslinked with TMPTA supported the adhesion, proliferation, and differentiation of dental pulp stem cells (DPSCs), providing a bioinstructive environment for tissue regeneration.^[^ ^14^ ^]^

Bis[2‐(methacryloyloxy) ethyl] phosphate (BMEP) is investigated in this study as an adhesive monomer. The phosphate group in BMEP enables self‐etching behavior by reacting with mineralized tissues, such as dentin, and interacting strongly with hydroxyapatite (HA), allowing effective demineralization, the formation of longer resin tags, and a stable hybrid layer.^[^ ^24^, ^25^ ^]^ This interaction promotes deeper etching compared to other adhesive systems like 10‐MDP, which primarily rely on superficial ionic bonding^[^ ^14^, ^16^, ^25^ ^]^ and enable an acid‐free etching procedure. The methacrylate groups of BMEP can react with thiol‐ene click crosslinking, promoting a high degree of polymerization to strengthen the adhesive network and enhance its mechanical properties.^[^ ^19^, ^26^ ^]^ Furthermore, BMEP acts as an effective matrix metalloproteinase (MMP) inhibitor, protecting the collagen matrix from enzymatic degradation and improving the long‐term durability of the resin‐dentin bond.^[^ ^25^, ^26^ ^]^ Additionally, compared to other etching systems,^[^ ^16^ ^]^ BMEP's ability to perform both chemical and micromechanical retention makes it superior in creating a robust adhesive interface.^[^ ^27^ ^]^

This study aimed to develop a biocompatible adhesive with strong dentin adhesion based on the multifunctional properties of TMPTA and BMEP, and with a potential for other mineralized tissues like bone. We hypothesized that incorporating BMEP into a TMPTA‐based thiol‐ene resin system would synergistically enhance mechanical strength, hybrid layer integrity, and biocompatibility in dentin bonding applications. The BMEP‐based resin system functions as a one‐step self‐etch adhesive, in which all components, like etching and bonding agents, are integrated into one solution and applied in a single step.

## 2 Experimental Section

Chemicals used in this study, Trimethylolpropane triacrylate (TMPTA), Trimethylolpropane tris(3‐mercaptopropionate) (TMPMP), Bis[2‐(methacryloyloxy)ethyl] phosphate (BMEP) and 2,2‐Dimethoxy‐2‐phenylacetophenone (DMPA), were all purchased from Sigma–Aldrich. All chemicals in experiments were used as received without further purification.

### 2.1 Material Fabrication

**Figure 1:**
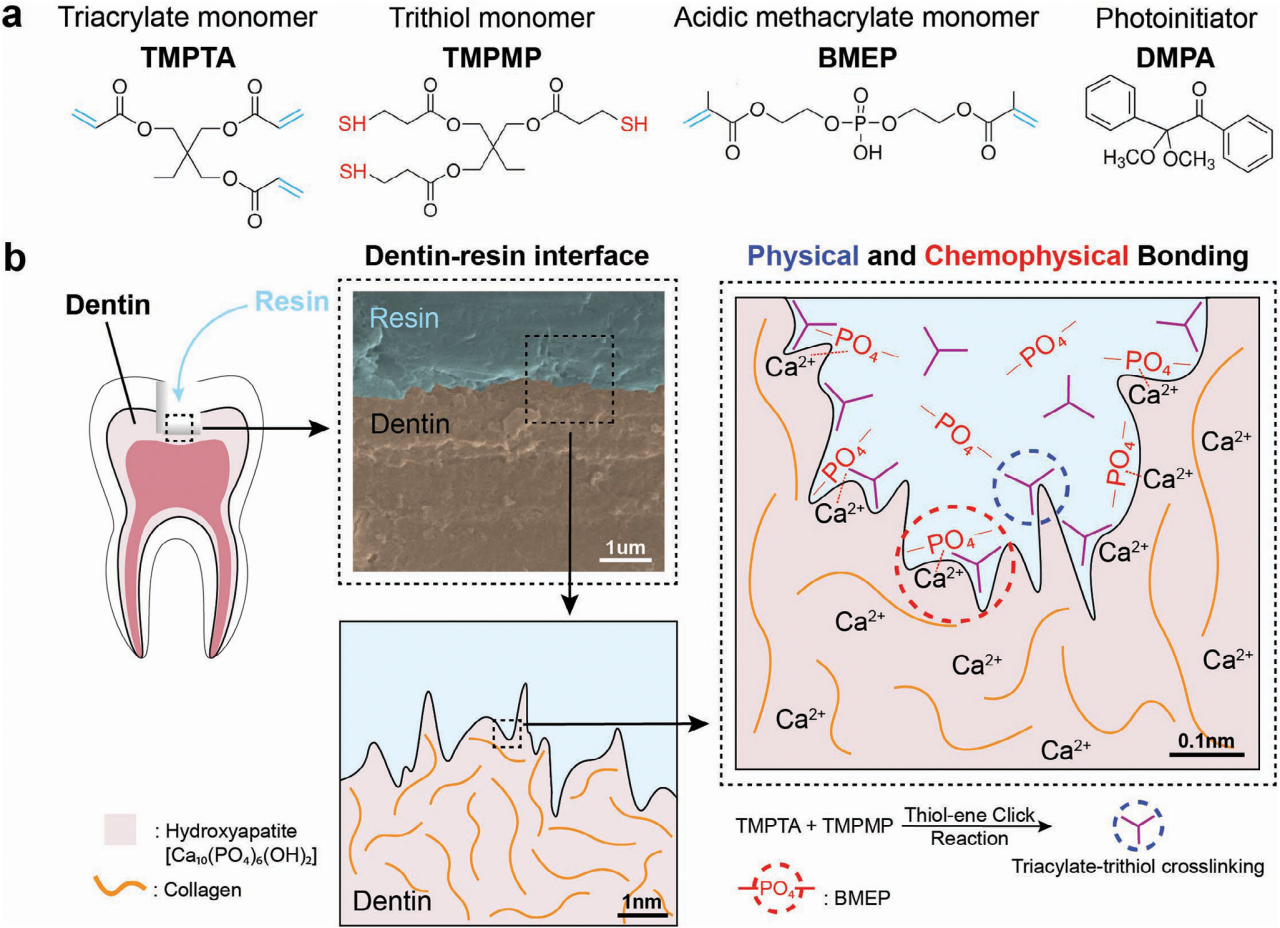
Illustration of TMPTA‐TMPMP resin‐dentin dual adhesion mechanisms. a) Overall schematic of resin with Trimethylolpropane triacrylate (TMPTA) and Trimethylolpropane tris(3‐mercaptopropionate) (TMPMP) as monomers, Bis[2‐(methacryloyloxy)ethyl] phosphate (BMEP) as the primer, and 2,2‐Dimethoxy‐2‐phenylacetophenone (DMPA) as the photoinitiator. b) Demonstration of both physical and chemophysical interlocks between resin and dentin through crosslinked polymers and chelation reactions involving BMEP.

The polymeric resin material was prepared using a dual monomer system consisting of TMPTA and TMPMP (**Figure** **1**a), utilizing thiol‐ene click chemistry (Figure S1, Supporting Information), which facilitates rapid polymerization through a radical‐mediated mechanism, and the photoinitiator, DMPA, was incorporated into the resin formulation to initiate the light‐curable reaction. A range of molar ratios of TMPTA and TMPMP was tested (1.5:1–5.5:1). 5% w/v BMEP was used for samples containing BMEP and 0.05% w/v DMPA was immediately added prior to shaker mixing for several minutes for homogenization. The solution was mixed at room temperature in a clean amber vial to prevent direct ambient light exposure. The BMEP‐based adhesive was formulated as a one‐step system, containing etching and bonding monomers, and no separate etching or priming step was performed.

### 2.2 Molecular Dynamics Simulation

Molecular dynamics (MD) simulations were performed using the Blend module in Materials Studio. TMPTA and TMPMP molecules were constructed and energy‐minimized using an appropriate force field. The Blend module was used to evaluate compatibility between the monomers based on calculated parameters, including binding energy, Flory–Huggins interaction parameter (χ), and phase behavior. Each molecule was minimized individually prior to blend simulations, which were run with TMPTA as the base and TMPMP as the screening molecule. Simulations were conducted under ambient temperature and pressure conditions, with up to 10 000 steps during the production phase. The results provided quantitative insights into interaction energies, coordination behavior, and compatibility profiles across different monomer ratios, aiding in the identification of optimal crosslinking conditions.

### 2.3 Oscillatory Shear Rheology

Rheology data were obtained using an HR30 Discovery Hybrid Rheometer (TA Inc.). Eighty microliters of the resin solution was dispensed onto the loading plate with the trim gap set to 1000 nm. Then, a time sweep of oscillatory shear rheology with a strain % of 10^−3^ and a frequency of 1.0 Hz was carried out at 25 °C for a duration of 930 s (30 s delay followed by 900 s of data collection). The power density of the ultraviolet (UV) source (Omnicure S2000, Excelitas) was 0.25 mW cm^−2^ with a 320–500 nm bandpass filter. The geometry gap was set to float to maintain a constant normal force. Polymerization shrinkage is measured by the change in gap during curing, defined by $\mathrm{Shrinkage}\;\%\;=\frac{\mathrm{original}\;\mathrm{gap}-\mathrm{final}\;\mathrm{gap}}{\mathrm{original}\;\mathrm{gap}}\times 100\%$.

### 2.4 Scanning Electron Microscopy (SEM)

Human third molars were obtained from the Penn Dental Oral Surgery clinic, following IRB exemption protocol #827807. Dentin samples were prepared by removing enamel from human molars using IsoMet Low Speed Saw, and the exposed dentin surface was sanded with 150 and 220 grit sandpapers. The resultant dentin samples had cuboid shapes, and the resin solution was pipetted onto one of the cuboid surfaces, which had the largest surface area. A cover glass treated with Smooth‐On Ease release spray was positioned over the resin solution to avoid oxygen inhibition. After being cured for 60 s under UV light with an intensity of 103.2 mW cm^−2^, the dentin sample coated with resin was detached from the cover glass. Two types of specimens were prepared based on the orientation of the dentinal tubules relative to the applied resin. For samples intended to visualize resin tags, the dentinal tubules were exposed mechanically by the previous polishing process. The resin was applied perpendicular to the tubule direction, and the cured samples were subsequently etched with 35% phosphoric acid to expose resin tag structures. For samples where resin was applied parallel to the tubules, only surface polishing (150 and 220 grit) was performed. Subsequently, SEM imaging of dentin samples was performed using Quanta 600 FEG ESEM.

### 2.5 Shear Bond Strength Test

Human third molars were obtained from the Penn Dental Oral Surgery clinic, following IRB exemption protocol #827807. Dentin surfaces were not acid‐etched prior to resin application. Instead, it was prepared using a 600‐grit polishing wheel (Buehler Ltd., Lake Bluff, IL, USA) and subsequently embedded in acrylic resin blocks. Resin solutions were applied to the dentin surfaces in cylindrical plastic molds and cured, with a contact area of ≈2.1 mm^2^. Clearfil SE Bond (Kuraray) was used as a commercial reference adhesive. It was applied according to the manufacturer's protocol: Primer was applied to the dentin surface for 20 s, gently air‐dried for 5 s, followed by Bond application, air‐thinning, and light‐curing for 10 s. All shear tests were carried out with a universal shear bond tester (BISCO Inc., Schaumburg, IL, USA) applying a force (N) parallel to the interface between the dentin and adhesive using notch method via a notched‐edge loading blade. Shear bond strength was calculated in megapascals using the formula: $\mathrm{shear}\;\mathrm{bond}\;\mathrm{strength}=\frac{\mathrm{load}\;(N)}{\mathrm{surface}\;\mathrm{area}\;(mm^{2})}$.

### 2.6 Nanoindentation Mapping

Nanoindentation was performed on a TI‐950 nanoindenter (Hysitron, USA) with a diamond Berkovich pyramidal tip. Hardness and modulus were measured as described by Oliver and Pharr,^[^ ^28^ ^]^ under load control, to a peak load of 8 mN, with a 5‐s loading time, 2‐s hold time at the peak load, and 5‐s unload time. A 25‐nm liftoff was used before testing to allow for proper surface detection,^[^ ^29^ ^]^ and the reduced elastic modulus was calculated from the unloading curve.^[^ ^28^ ^]^ Testing was performed on the dentin‐resin interfaces, with the indentation direction parallel to the interfacial plane. The interface was exposed by polishing the cured dentin‐resin samples with 150 and 220 grit sandpapers, following the same protocol used for SEM sample preparation. A testing array was constructed to measure the elastic modulus of the dentin and resin as a function of the distance from the interface.

### 2.7 Cell Culture

BJ cells were purchased from ATCC. Human dental pulp stem cells (hDPSCs) were purchased from Lonza Bioscience (Walkersville). Low‐glucose Dulbecco's Modified Eagle Medium was purchased from Gibco. Fetal bovine serum (FBS) was purchased from Cytiva. Penicillin‐streptomycin (P/S) was purchased from Thermo Fisher Scientific. Bovine FGF basic protein (bFGF) was purchased from Fisher Scientific.

BJ cells were cultured in low‐glucose Dulbecco's Modified Eagle Medium (DMEM) supplemented with 10% FBS and 0.1% bFGF. hDPSCs were cultured in low‐glucose Dulbecco's Modified Eagle Medium (DMEM) supplemented with 10% FBS, 1% P/S and 0.1% bFGF. All cell lines were grown at 37 °C under a 5% CO_2_ humidified atmosphere until confluence.

### 2.8 Biocompatibility Test

Resin samples with and without BMEP were submerged in 1mL (material:solution ratio = 0.785 cm^2^·mL^−1^ low‐glucose Dulbecco's Modified Eagle Medium (DMEM, Gibco) at 37 °C with 5% CO_2_ for 7 days. Collected conditioned media were diluted with low‐glucose DMEM to a final concentration of 100%, 50%, and 25% of the original conditioned media. Human dental pulp stem cells (hDPSCs) and BJ cells (human foreskin fibroblast) were seeded at 62 500 cells·cm^−2^ in a 96 well‐plate and incubated in low glucose DMEM with 10% fetal bovine serum separately. After 24 h, cell culture media were replaced by 100 µL serially diluted original condition media, and cells were kept cultured for 24 h. Cells replaced with 100 µL low glucose DMEM and kept culturing for 24 h as nontreated control. For fluorescence imaging, cells in conditioned media (100%, 50%, and 25%), and nontreated cells were fixed in 2% paraformaldehyde (PFA) for 30 min and then stained with Phalloidin and 4′,6‐diamidino‐2‐phenylindole (DAPI). For cell viability experiments, released lactate dehydrogenase (LDH) in cell culture supernatants was measured by CytoTox 96 Nonradioactive Cytotoxicity Assay (Promega). Relative cell viability was compared with the maximum LDH release control. For cell counting experiments, cells were trypsinized by Trypsin‐EDTA (ethylenediaminetetraacetic acid, 0.05%, Gibco) and retrieved from 96 plate. Retrieved cells were stained with a 0.4% solution of trypan blue for counting.

### 2.9 Statistical Methods

GraphPad Prism was used for statistical analysis and data visualization. Rheology data were analyzed with unpaired *t*‐test. Shear bond test data was analyzed with Brown–Forsythe and Welch ANOVA test. Sample size and p‐values are noted in the figures. Cell viability and cell counting data were analyzed with a 2‐way ANOVA test.

## 3 Results

The multifunctional resin material features physical and chemophysical adhesion mechanisms for bonding with mineralized tissues like dentin (Figure 1). The roughness and unevenness of the dentin surface provide micromechanical, physical bonding between the triacylate‐trithiol network and micro‐roughness on the interface. Monomer BMEP provides chemophysical bonding by chelation of calcium ions in hydroxyapatite of dentin by the phosphate group in BMEP, which has been previously shown in other studies.^[^ ^30^ ^]^ BMEP can also provide increased roughness for physical bonding by etching the surface.^[^ ^25^ ^]^ Therefore, BMEP enables a one‐step resin adhesive system.

**Figure 2:**
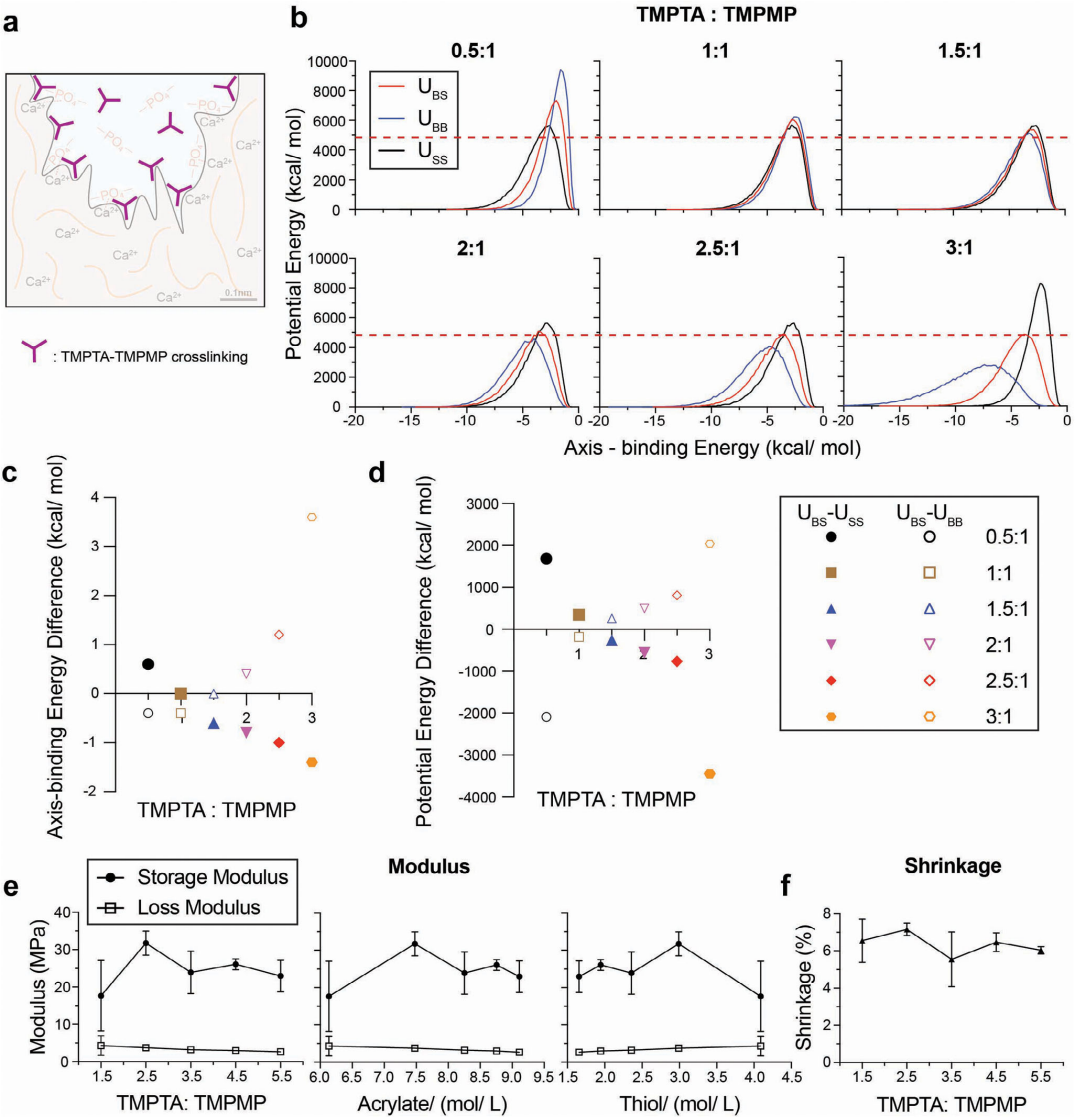
Molecular dynamics simulations and comparative modulus and shrinkage analysis for resin systems with varying compositions of TMPTA and TMPMP. a) Illustration of TMPTA‐TMPMP crosslinking in resin. b) Molecular dynamics simulation results of potential energy and axis‐binding energy profile for TMPTA and TMPMP reacting under different ratios. U – potential energy; S – screening material, TMPMP; B – base material, TMPTA. c,d) Summary plots for (c) axis‐binding energy difference and (d) potential energy difference between U_BS_ and U_SS_ or U_BB_ for different TMPTA: TMPMP ratios. e) Storage modulus and loss modulus of resin plotted as a function of: TMPTA: TMPMP ratios, acrylate concentration, and thiol concentration. f) Shrinkage of resin under different TPMTA: TMPMP ratios.

Cross‐linked polymer networks reinforce the physical bonding of resin to dentin and increase the cohesive strength of the material (**Figure** **2**a). The cross‐linking of TMPTA polymers was investigated by molecular dynamics (MD) simulations and oscillatory rheology. MD calculations of TMPTA and TMPMP simulated the resin materials at the molecular level to predict polymerization dynamics and crosslinking efficiency.^[^ ^31^ ^]^ The red solid curves show the energy between base materials and screening materials (U_BS_), the blue solid curves are the energy between base materials and base materials (U_BB_), and the black dotted curves represent the energy between screening materials and screening materials (U_SS_) (Figure 2b). The results were evaluated to identify the appropriate reacting ratios by 1) minimizing U_BS_, 2) aligning interaction energies, and 3) finding the peak of U_BS_ in between of U_SS_ and U_BB_. U_BS_’s potential energy is relatively low with molar ratios of 1.5:1 to 3:1. Lower potential energy of U_BS_ in atomistic simulations should indicate the mechanical stability of the resin.^[^ ^31^ ^]^ The red dashed lines indicate the lowest U_BS_ peak potential energy across six ratios. With increasing TMPTA and TMPMP molar ratios, the axis‐binding energy difference between U_BS_ and U_SS_ decreased while the difference between U_BS_ and U_BB_ increased (Figure 2c). Ratios ranging from 0.5:1 to 2.5:1 show both values near zero. U_SS_, U_BB_ and U_BS_ should have similar curve shapes, and their axis should approximately align with each other (namely, their axis's position should have the same axis‐binding energy value on x‐axis). Aligning the interaction energies of different monomers in thiol‐ene systems optimizes polymerization dynamics by facilitating molecular collisions along favorable reaction pathways.^[^ ^32^ ^]^ The potential energy difference between U_BS_ and U_SS_ decreased and the difference between U_BS_ and U_BB_ increased (Figure 2d). The energy difference was largest with ratios at 0.5:1 and from 2.5:1 to 3:1. The peak of U_BS_ should be in between U_SS_ and U_BB_ and the one with larger energy differences is preferred because energy differentials between interacting components are critical for optimizing crosslinking reactions as larger energy differences drive more efficient polymerization.^[^ ^19^ ^]^ Together, these results suggest that TMPTA: TMPMP ratio at 2.5:1 is most favorable for the thiol‐ene reaction. The predicted molecular structure is shown in Figure S2 (Supporting Information).

Oscillatory shear rheology was performed on resins with TMPTA: TMPMP ratios ranging from 1.5: 1 to 5.5: 1 with or without BMEP. It revealed that the resin system exhibited a maximum storage modulus of ≈32 MPa at a TMPTA: TMPMP molar ratio of 2.5:1 (7.5 mol L^−1^ TMPTA and ≈2.9–3.0 mol L^−1^ TMPMP), with shrinkage remaining stable at ≈6% across all ratios (Figure 2e,f). These data showed that increasing acrylate concentration above 7.5 mol L^−1^ (ratio = 2.5) resulted in a trend of decreasing storage modulus, which is consistent with the MD simulations in Figure 2b–d. Addition of BMEP (5 wt%) slightly increased the storage modulus across all ratios (23–36 MPa with BMEP vs. 17–32 MPa without), without significantly affecting the loss modulus (≈3.5 MPa) or polymerization shrinkage (≈6%) (Figure S3, Supporting Information). These values are comparable to other resin systems based on thiol‐ene click chemistry^[^ ^19^, ^33^, ^34^ ^]^ and polymerization shrinkage studies (1–6%).^[^ ^35^, ^36^, ^37^ ^]^

We investigated the mechanical and adhesive properties of the triacrylate/trithiol resin (TMPTA: TMPMP = 2.5) at the dentin‐resin interface. Nanoindentation was performed on a thin film of TMPTA/TMPMP resin to evaluate the homogeneous curing and nanomechanical properties. The thiol‐ene TMPTA and TMPMP radical‐initiated propagation rate constant was estimated to be nearly 1000 times higher than that of methacrylate‐methacrylate groups,^[^ ^31^ ^]^ which leads to a rigid and homogeneous covalently crosslinked polymer network. This homogeneity is verified through the nanoindentation modulus in Figure S4 (Supporting Information). It shows the modulus was uniformly ≈1 GPa across the resin film's frontside (0.992 GPa) and backside (1.125 GPa). The slight difference in modulus observed here is attributed to the longer‐time oxygen exposure (order of seconds) on the resin droplet's surface before sealing by the top glass slide, causing more oxygen inhibition.^[^ ^38^ ^]^

**Figure 3:**
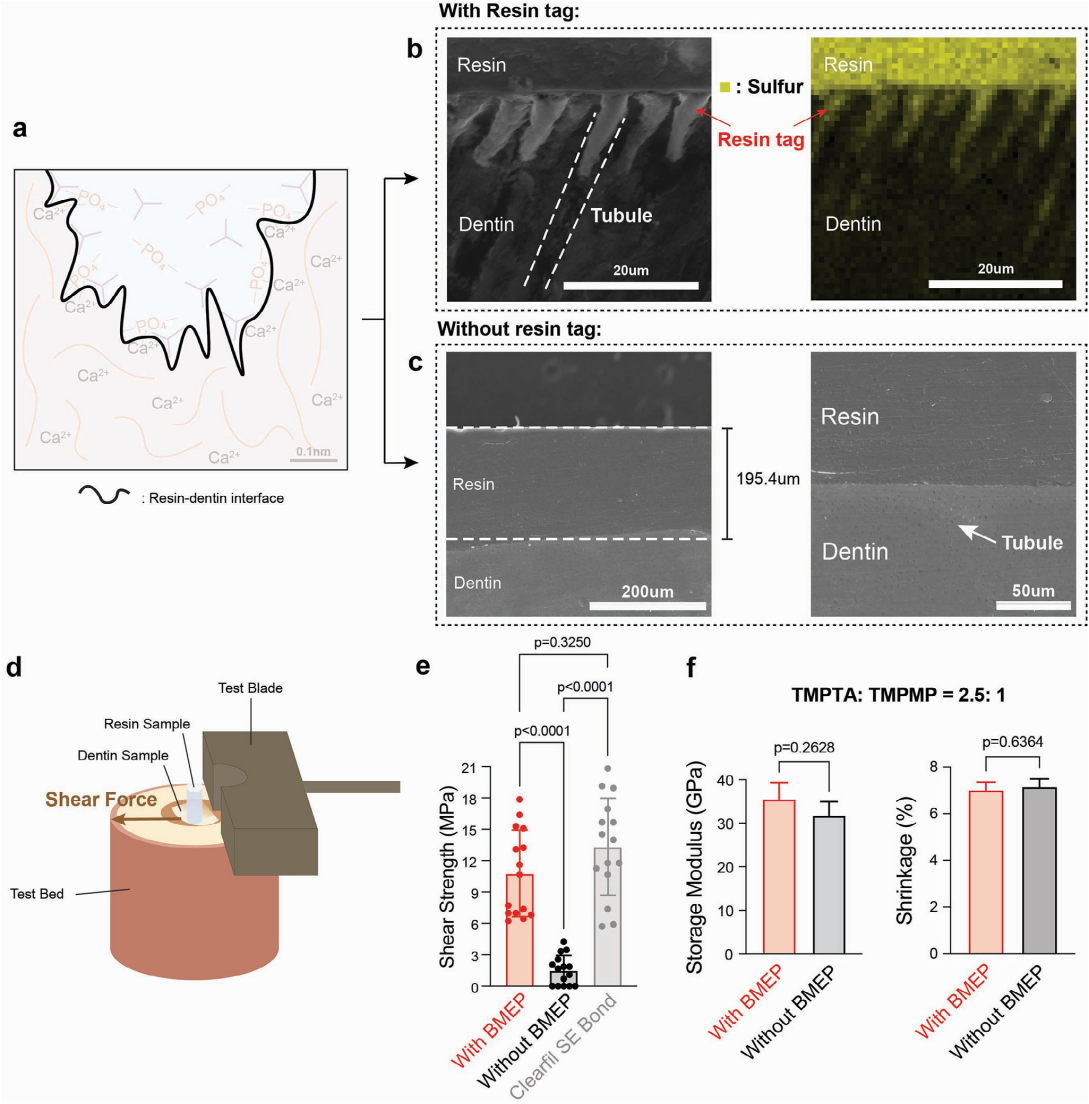
Comparative adhesion, storage modulus and shrinkage analysis of the resin‐dentin interface and resin systems with and without BMEP. a) Illustration of the resin‐dentin interface. b) SEM (left) and EDS (right) images of the dentin interface (resin applied perpendicular to dentinal tubules) with the resin tag exposed. (Yellow: sulfur). c) SEM images of the dentin interface (resin applied parallel to tubules) without resin tag under 200 and 50 µm scale. d) Shear test demonstration for adhesion situation between resin and dentin sample (contact area = 2.1 mm^2^), and e) corresponding shear strength statistical results for resins and the commercial primer Clearfil SE Bond (Brown–Forsythe and Welch Test, sample size *n* = 15). f) Statistical results of two resin systems about storage modulus and shrinkage percentage (unpaired t‐test, sample size n = 3).

The adhesive interface between the human dentin and the resin material was characterized by SEM, EDS, shear bond strength, and shear rheology (**Figure** **3**). The resin was cured onto dentin surfaces with exposed dentinal tubules. SEM imaging (Figure S5, Supporting Information) showed resin tags in dentinal tubules, which were confirmed by the sulfur elemental analysis (yellow) (Figure 3b). When cured onto smooth, polished dentin surfaces, SEM imaging on the resin layer (196 µm thick) showed a smooth interface with dentin with no defects, suggesting an intact micromechanical bonded interface (Figure 3c). Shear bond tests were performed on smooth, polished dentin substrates (Figure 3d). The average shear strength of resin materials with and without BMEP was 10.8 and 1.5 MPa, respectively (Figure 3e), compared to 13.3 MPa with the commercial primer Clearfil SE Bond. The adhesive strength of the multifunctional resin with BMEP showed no statistically significant difference with Clearfil SE Bond, which was consistent with the reported shear bond strength of other commercial adhesive resin systems.^[^ ^39^ ^]^ Clearfil SE Bond was selected as a benchmark due to its widespread clinical use and its phosphate‐containing 10‐MDP methacrylate chemistry. This comparison enables a direct evaluation of bonding performance between two phosphate‐containing adhesive systems. Methacrylate‐based systems are known to exhibit low double bond conversion, residual monomer release, and network heterogeneity.^[^ ^5^, ^13^, ^14^ ^]^ In contrast, the triacrylate‐trithiol resin forms a more uniform crosslinked network, potentially improving biocompatibility without compromising bond strength. Further, the modulus and shrinkage comparison of resin materials with or without BMEP did not show a statistically significant difference at a molar ratio of TMPTA: TMPMP = 2.5 (Figure 3f). These data suggest that the addition of BMEP significantly strengthens the adhesion on the resin‐dentin interface without negatively impacting the resin system's mechanical properties.

**Figure 4:**
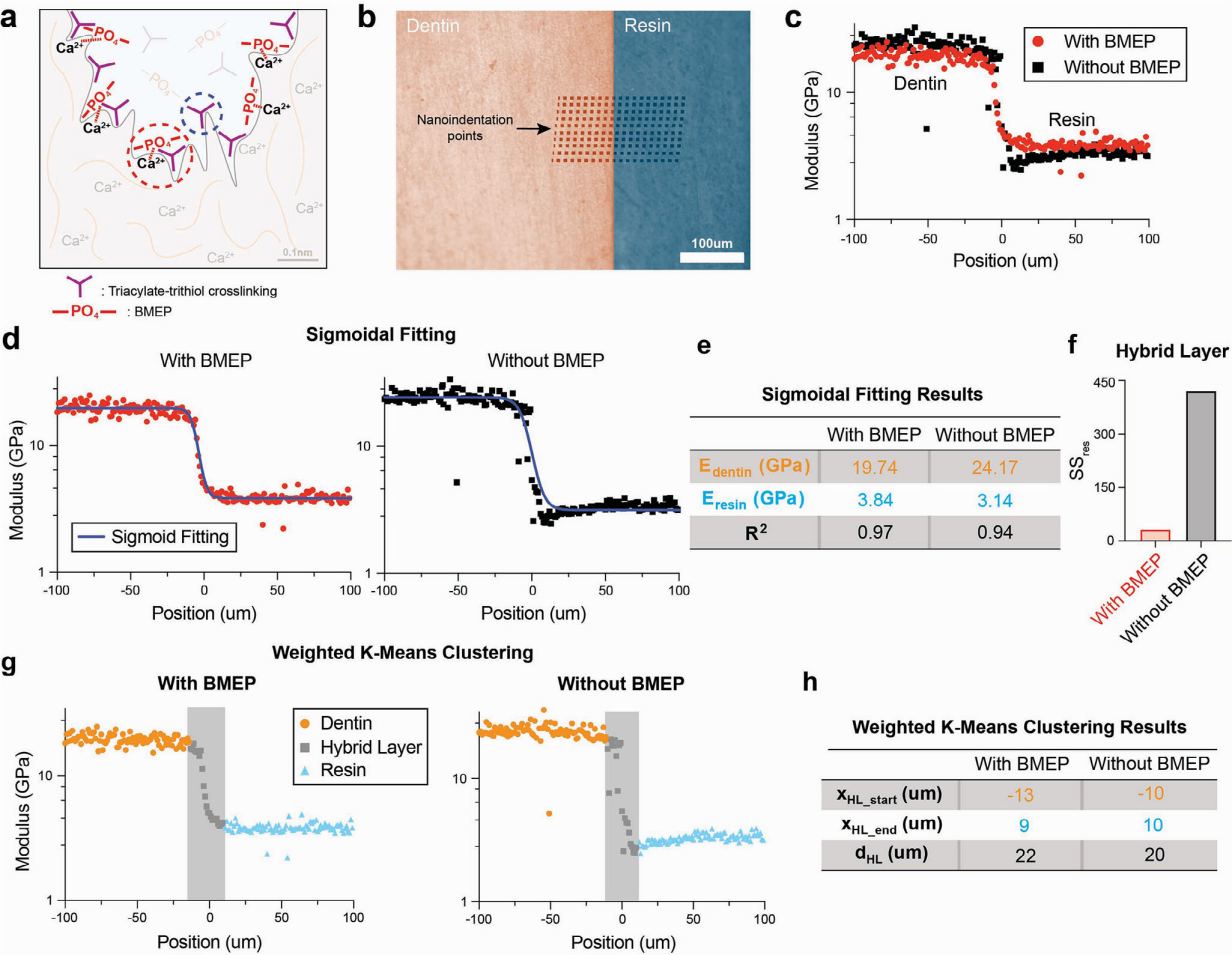
Nanomechanical analysis of the resin‐dentin adhesive interface. a) Illustration of two interlocking mechanisms on the resin‐dentin interface. b) Nanoindentation mapping array (10 sperate indentation lines * 20 indentation tests), c) modulus results for resin with (red) and without (black) BMEP, plots for (d) sigmoidal fitting results (blue curves), e) corresponding summary table (E: modulus, R^2^: the coefficient of determination), and f) comparison on HL's sum of residual (SS_res_). g) Weighted k‐means clustering results (dentin region – orange, hybrid layer (HL) region – grey, resin region – blue), and h) corresponding summary table (x_HL_start/ end_: hybrid layer region starting/ ending position, d: thickness). All the results demonstrated here represent the aggregate outcomes derived from the analysis of all ten individual indentation line tests.

We hypothesized that the phosphate‐hydroxyapatite chemophysical interactions of BMEP reinforce the nano‐mechanical interface of resin and dentin (**Figure** **4**). Nanoindentation line mapping was performed to investigate the nanomechanical properties of the dentin‐resin interface and its hybrid layer with a line of 20 indentation tests (black dots), spaced 10 µm apart, beginning in the dentin 100 µm away from the interface and running to 100 µm inside the resin (Figure 4b). Indents were spaced by a distance of 10 µm to avoid any interference from subsurface plastic deformation from neighboring indents.^[^ ^29^ ^]^ Improved spatial resolution across the interface of 1 µm was achieved by running 10 separate lines of indents, each beginning 1µm closer to the interface, with each line being spaced 10 µm apart. This resulted in a total array of 200 indents on each specimen and an indentation test every 1 µm away from the interface from 100 µm inside the dentin to 100 µm inside the resin.

Line mapping nanoindentation results of TMPTA/TMPMP resin samples with BMEP (red dots) and without BMEP (black squares) are shown in Figure 4c. Sigmoidal fitting was utilized to model the transition between dentin and resin modulus (Figure 4d) because a similar sigmoidal shape was observed in nanoindentation studies of the dentin‐enamel junction.^[^ ^40^, ^41^ ^]^ The sigmoidal expression to describe the relationship between modulus and indentation position is $E\;=\frac{E_{\mathrm{resin}}-E_{\mathrm{dentin}}}{1+e^{k(x-x_{0})}}+E_{\mathrm{dentin}}$. E represents modulus, k is a fitting parameter, x is the position, and x_0_ is the shifted origin. The fitting parameter results in Figure 4e show a 22% higher modulus (3.84 GPa, R^2^ = 0.97) with BMEP compared to without BMEP (3.14 GPa, R^2^ = 0.94). Further, Figure 4f shows that BMEP improved the sigmoidal transition of modulus from dentin to resin, quantified by a lower sum of squared residuals (SS_res_) within the hybrid layer (32.6), compared to resin without BMEP (422.4). Weighted k‐means clustering analysis^[^ ^42^ ^]^ was performed to calculate the thickness of the hybrid dentin‐resin layer (Figure 4g – dentin (orange), hybrid layer (grey), resin (blue)). Resin materials with BMEP show a hybrid layer thickness of 22 µm, compared to 20 µm for samples without BMEP (Figure 4h). This hybrid layer thickness is larger than the numbers reported for other resin systems, which range from 0.3 to 10 µm.^[^ ^43^, ^44^, ^45^, ^46^ ^]^ These results suggest the addition of BMEP increases the thickness of the hybrid layer by 10%, which improves the mechanical stability of the dentin‐resin interface. This is consistent with the shear strength results and other studies that suggest the importance of reinforcing the hybrid layer.^[^ ^43^, ^47^ ^]^ This nanoindentation result shows consistency in a more stabilized hybrid layer between materials to enhance bonding strength.^[^ ^44^ ^]^

**Figure 5:**
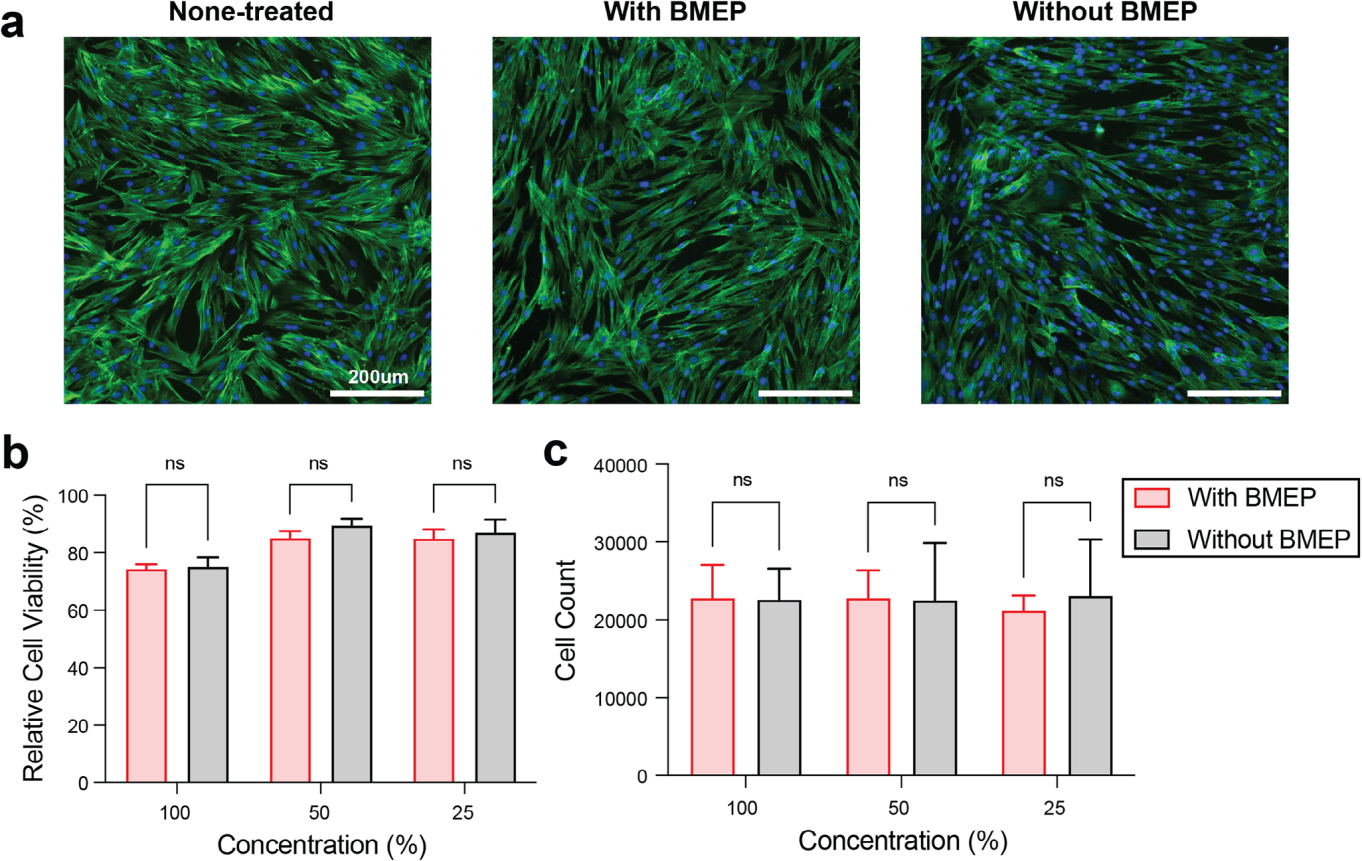
Biocompatibility of resin systems with and without BMEP on hDPSCs. a) Fluorescence imaging (scale bar 200 µm) of hDPSCs after 24 h culture in original condition (100% concentration) media compared to negative control, stained for nuclei (blue) and F‐actin (green). b) Relative cell viability, compared to negative control, and c) cell counts of hDPSCs after 24 h culture in condition media. Condition media are diluted with DMEM to a total concentration of 100%, 50%, and 25% of the original condition media. *n* = 3 biological replicates, error bars represent SD.

Biocompatibility tests were performed using resin‐treated conditioned media to confirm that the triacrylate‐trithiol polymer adhesive system supports cell viability^[^ ^14^ ^]^ (**Figure** **5**; Figures S6 and S7, Supporting Information). Fully cured resin samples with and without BMEP were conditioned in low‐glucose DMEM at 37 °C. Media were collected after 7 days to treat human dental pulp stem cells (hDPSCs) and BJ fibroblast cells. Cell morphology was imaged after 24 h exposure to original condition media by fluorescence imaging of cells stained for cell nuclei (DAPI, blue) and F‐actin (phalloidin, green) (Figure 5a; Figures S6 and S7a, Supporting Information). The cytotoxicity of resin materials was measured by LDH release after 24 h exposure to serially diluted original condition media. In the 100% condition media group, the relative hDPSC viability of resin with BMEP was 74.35%, and the resin without BMEP was 75.17%. In the 50% and 25% condition media group, a slight increase in the relative cell viability of both resin systems was shown, with 85.09% and 84.94% viability for the resin with BMEP, respectively, compared to 89.58% and 87.01% for the resin without BMEP (Figure 5b). In the 100% condition media group, the relative BJ cell viability of resin with BMEP was 83.24%, and the resin without BMEP was 80.97%; in the 50% and 25% condition media group, the relative cell viability of resin with BMEP was 86.33% and 86.39%, respectively, compared to 88.54% and 87.71% for the resin without BMEP (Figure S7b, Supporting Information). These results revealed low cytotoxicity of this triacrylate‐trithiol polymer system in dental tissues and no significant differences between the resins with and without BMEP. hDPSCs and BJ cells were retrieved, respectively, and cell numbers in each condition were counted after 24 h exposure to the serially diluted condition media. Media containing released residuals of resin materials with and without BMEP did not significantly affect cell number and proliferation in both hDPSCs and BJ cells (Figure 5c; Figure S7c, Supporting Information). Together, we confirmed that TMPTA resin systems had low cytotoxicity, and resins with and without BMEP didn't exhibit significant differences in their biocompatibility. This finding can be applied to incorporate additional functional groups for drug delivery and remineralization at the mineralized tissue interface.

## 4 Conclusion

This study demonstrates the mechanical and adhesive properties of a multifunctional polymeric resin system composed of TMPTA and BMEP, designed to improve adhesion to mineralized tissues. Molecular dynamics simulations identified 2.5:1 TMPTA: TMPMP as the optimal ratio for the thiol‐ene polymerization reaction, which was experimentally validated through rheological and mechanical testing. The addition of BMEP in the TMPTA resin played a crucial role in enhancing adhesion strength, achieving a shear bond strength of 10.8 MPa, comparable to commercial primer Clearfil SE Bond, without significantly affecting biocompatibility or shear modulus. Nanoindentation mapping further revealed that BMEP increased dentin‐resin hybrid layer thickness with a sigmoidal modulus profile. Biocompatibility tests demonstrated that the TMPTA resin materials with and without BMEP had low cytotoxicity. The combination of micromechanical interlocking and chemophysical bonding with a thiol‐ene polymer network enables strong adhesion while maintaining biocompatibility. Overall, the TMPTA‐TMPMP‐BMEP resin system offers a promising strategy to enhance the longevity and clinical performance of dental adhesives. Its modular and multifunctional design supports integration with bioactive approaches, such as remineralization and localized drug delivery, highlighting its translational potential not only in regenerative dentistry but also in broader biomedical applications, including bone fixation and tissue‐interfacing coatings.

## Conflict of Interest

Kyle Vining is a co‐inventor of a issued patent related to this paper ‐ US11224679B2.

## Author Contributions

Y.L., K.T.T., and K.H.V. contributed to the conceptualization, data curation, writing, and review and editing of this manuscript. Y.L., C.Z., S.F., J.L., K.C., Y.J., and O.F. contributed to the data collection and analysis. Y.L., R.X., and O.A.T. contributed to the protocol formation. Y.H. and L.R. contributed to the data modeling and visualization. All authors contributed to the review and editing.

## Supporting information



Supporting Information

## Data Availability

The data that support the findings of this study are openly available in Dyrad at https://doi.org/10.5061/dryad.hmgqnk9ws, reference number 1.
