# Supplementary material for: Biocompatible Multifunctional Polymeric Material for Mineralized Tissue Adhesion
Source: Adv Healthc Mater. 2025 Aug 18;14(27):e01993. doi: 10.1002/adhm.202501993 (PMC12538523; doi:10.1002/adhm.202501993)
Supplement: Supplementary file 1 — Supporting Information [file ADHM-14-0-s001.docx]

# **Supplemental Information**

**Biocompatible Multi-functional Polymeric Material for Mineralized Tissue Adhesion**

Yan Luo^1^, Chenyang Zhang^2^, Sage Fulco^1^, Jingyi Liu^3^, Keyu Chen^2^, Yuntao Hu^4, 5^, Yuchen Jiang^2^, Rui Xu^6^, Leela Rakesh^7^, Ozer Fusun^8^, Ottman Tertuliano^1^, Kevin Turner^1, 2^, Kyle H. Vining^2, 8, 9 *^

1 Mechanical Engineering and Applied Mechanics, School of Engineering and Applied Science, University of Pennsylvania, Philadelphia, PA, United States

2 Materials Science and Engineering, School of Engineering and Applied Science, University of Pennsylvania, Philadelphia, PA, United States

3 Bioengineering, School of Engineering and Applied Science, University of Pennsylvania, Philadelphia, PA, United States

4 Robotics, School of Engineering and Applied Science, University of Pennsylvania, Philadelphia, PA, United States

5 Computer and Information Science, School of Engineering and Applied Science, University of Pennsylvania, Philadelphia, PA, United States

6 Biomedical Engineering, School of Engineering, Vanderbilt University, Nashville, TN,

United States

7 Mathematics, Center for Applied Mathematics and Polymer Fluid Dynamics, College of Science and Engineering, Central Michigan University, Mount Pleasant, MI, United States

8 Preventive and Restorative Dentistry, School of Dental Medicine, University of Pennsylvania, Philadelphia, PA, United States

9 Center for Innovation and Precision Dentistry, University of Pennsylvania, Philadelphia, PA, United States

*Corresponding Author, [viningk@upenn.edu](mailto:viningk@upenn.edu)

**Nanoindentation Test**

The resin film was crafted by pipetting 100μL of resin solution onto one MTC Bio microscope slide with a 25*75mm dimension. This initial layer was then complemented by carefully stacking another slide in a cross formation, creating a square area (25*25mm) with the resin solution sandwiched between them. Next, a UV light with an intensity of 103.2mW/cm² was directed onto the square area for a duration of 60 seconds using a UV lamp. Once the resin film had undergone complete curing, it was gently peeled off from the slides, designating the side where the light entered as the front side, and the side where the light exited as the back side. Subsequently, seven different points, spaced at 8 mm intervals, were chosen on both sides for the nanoindentation test.

Nanoindentation experiments were carried out using KLA iMicro Nano indenter with a Berkovich tip and the advanced energy and hardness (E&H) method^1^. This methodology involved setting specific parameters: a target load of 10 mN, a target depth of 1000 nm, and a target indentation strain rate of 0.1/s. All tests were carried out at room temperature (22–24 °C).

# **Supplemental Figures**


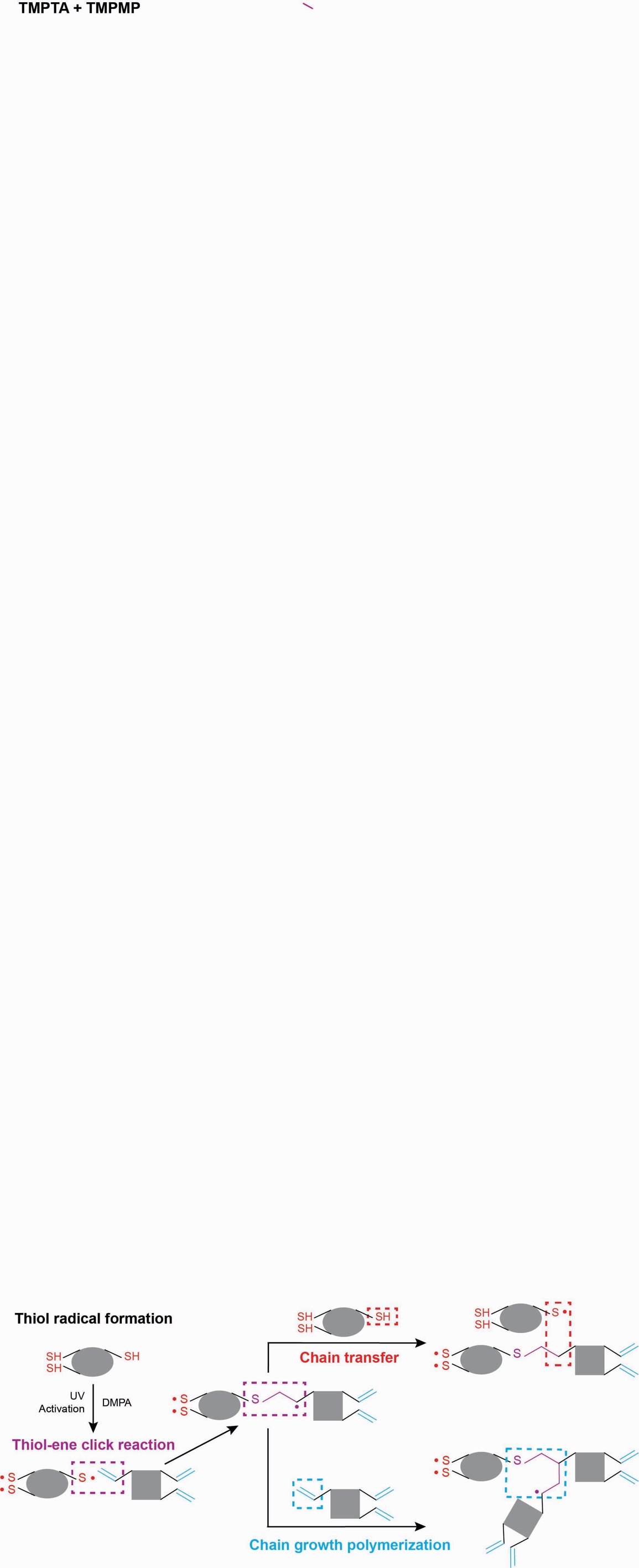


Figure S1. Thiol-ene click reaction processes for crosslinking TMPTA and TMPMP.


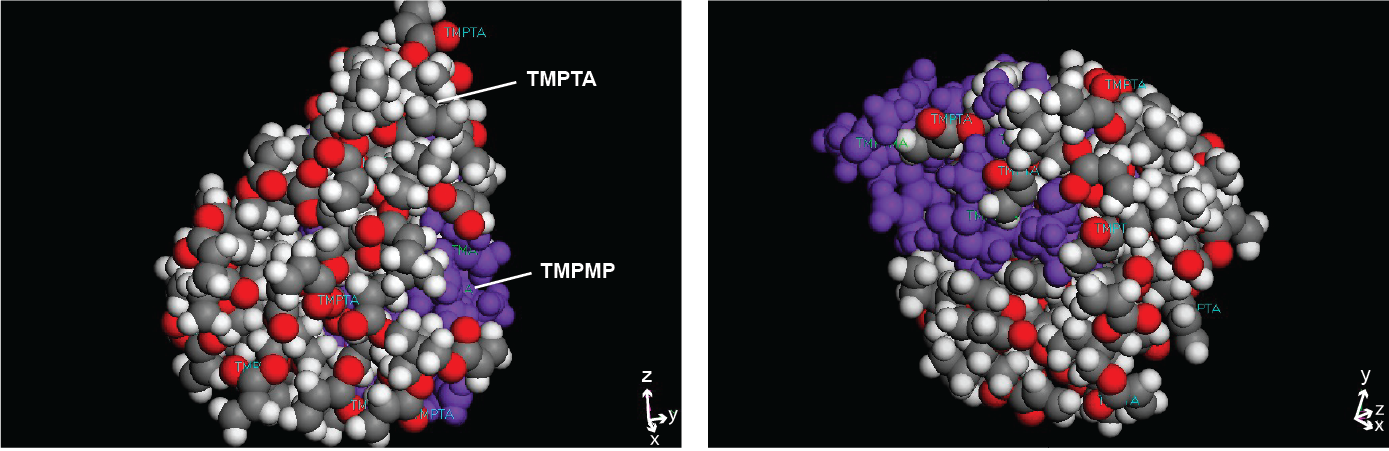


Figure S2. The predicted molecular structure of the dental resin material with the five TMPTA molecules and two TMPMP molecules. TMPTA: red – oxygen atom, grey – carbon atom, white – hydrogen atom; purple – TMPMP molecule.


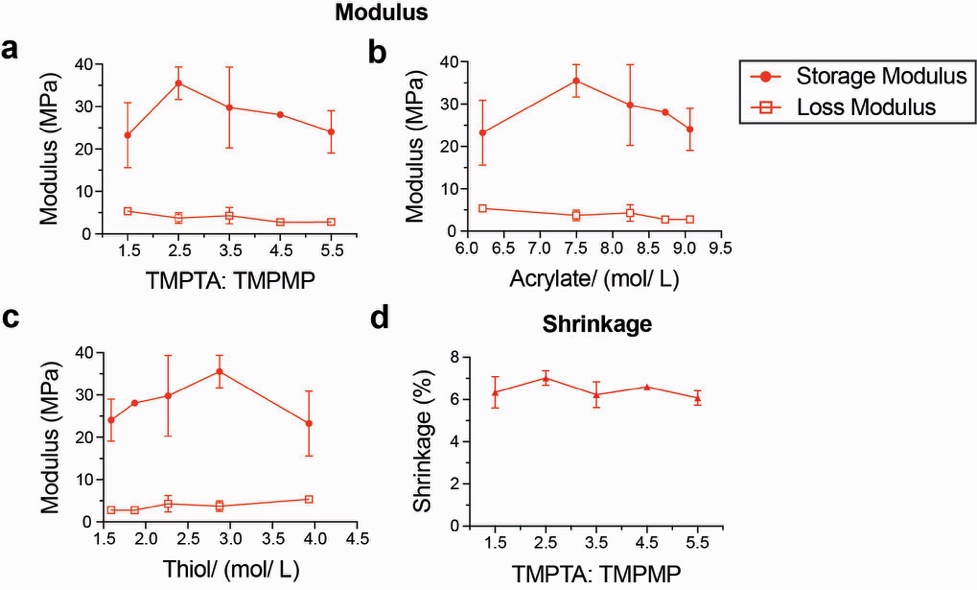


Figure S3. Storage modulus and loss modulus of resin with BMEP plotted as a function of: (a) TMPTA: TMPMP ratios, (b) acrylate concentration and (c) thiol concentration. (d) Shrinkage of resin with BMEP (5 wt%) under different TPMTA: TMPMP ratios.


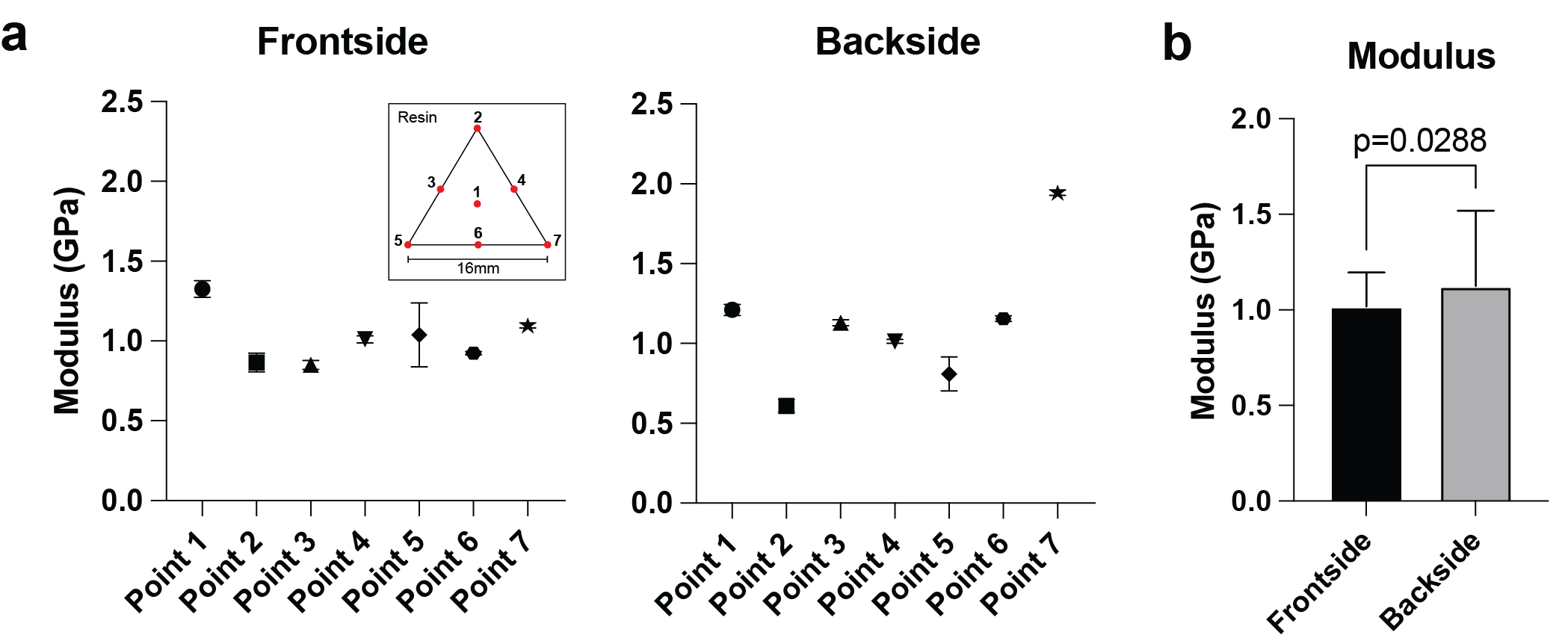


Figure S4. (a) Modulus data from nanoindentation on thin resin film’s frontside and backside. Inset: illustration for nanoindentation positions of 7 points. (b) Statistics analysis of t test on modulus data of frontside and backside (sample size=77).


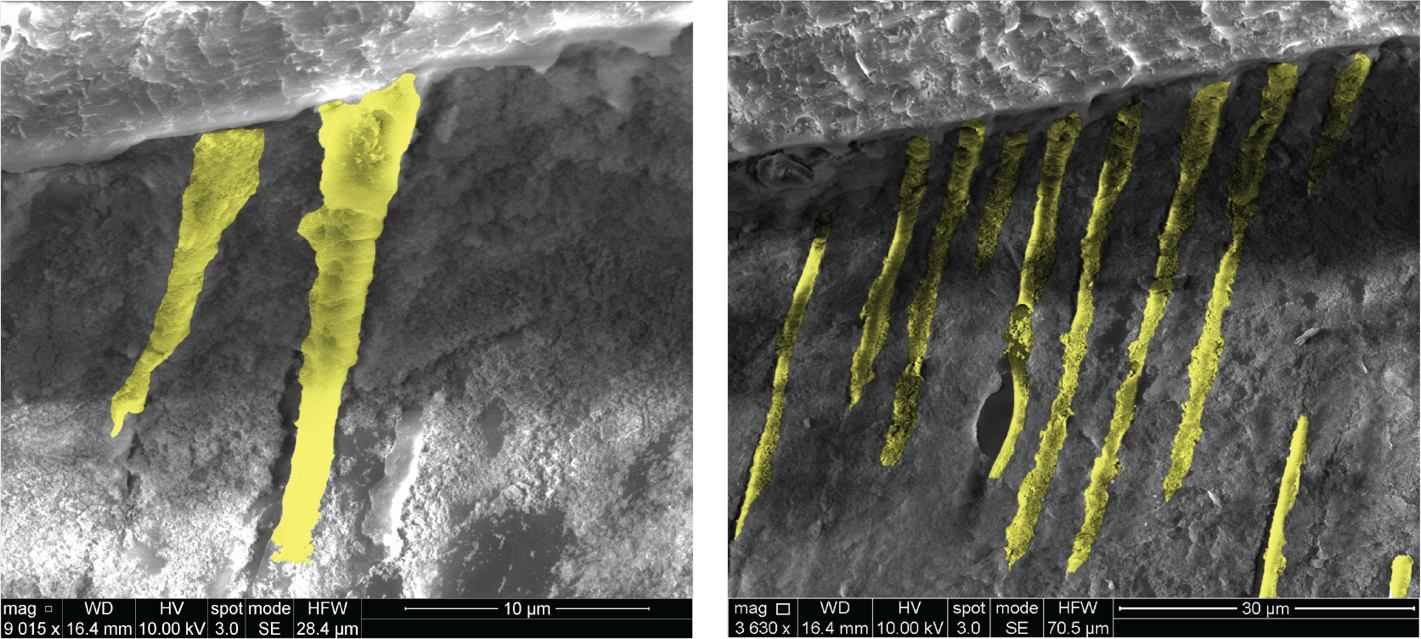


Figure S5. SEM images of along-tubule-resin-coated dentin interface with resin tag (yellow) exposed under 10 $\mu$m and 30 $\mu$m scale.


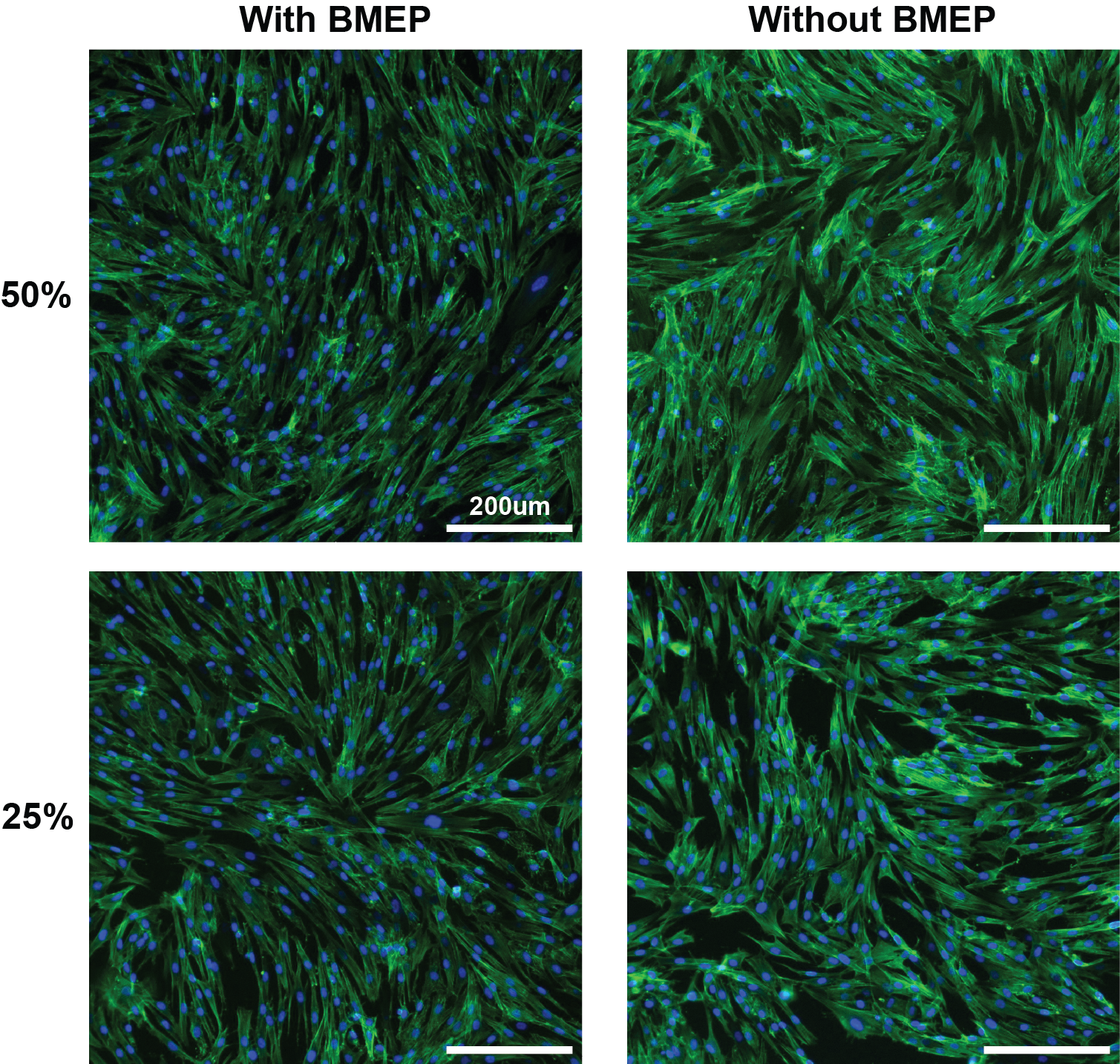


Figure S6. Fluorescence imaging of hDPSCs treated 24 hours with conditioned media (50 and 25% dilutions) from resin samples with or without BMEP. F-actin (green, phalloidin) and nuclei (blue, DAPI). Scale bar 200 $\mu$m.


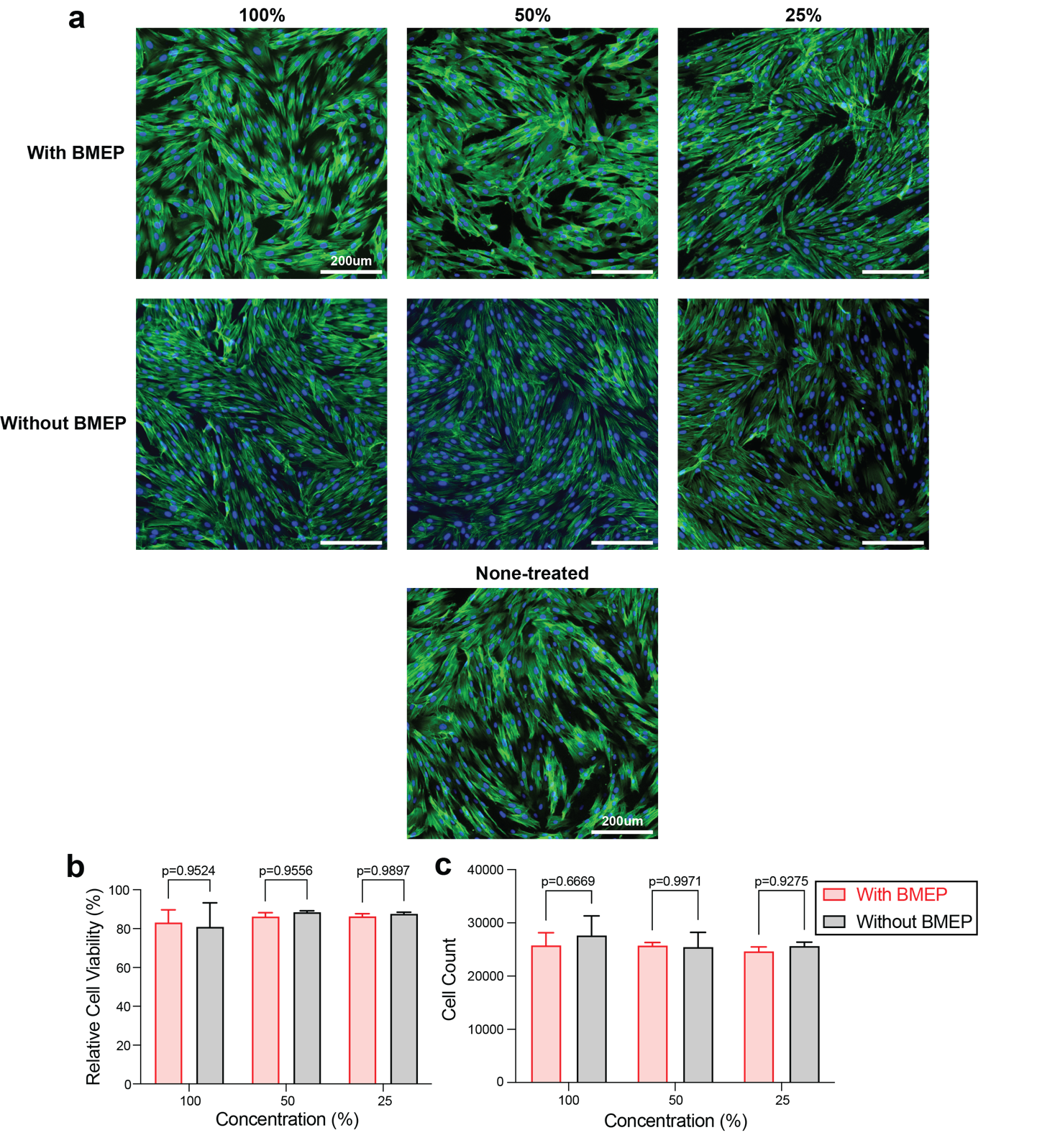


Figure S7. Biocompatibility of resin systems with and without BMEP on BJ fibroblasts. (a) Fluorescence imaging (scale bar 200 $\mu$m) of BJ cells after 24 h culture in original condition (100%, 50% and 25% concentration) media compared to negative control, stained for nuclei (blue) and F-actin (green). (b) Relative cell viability, compared to negative control, and (c) cell counts of BJ cells after 24 h culture in condition media. Condition media are diluted with DMEM to a total concentration of 100%, 50% and 25% of the original condition media. n = 3 biological replicates, error bars represent SD.

# References

(1) Oliver, W. C.; Pharr, G. M. Measurement of hardness and elastic modulus by instrumented indentation: Advances in understanding and refinements to methodology. Journal of materials research 2004, 19 (1), 3-20.
